# Association of Increased Circulating Acetic Acid With Poor Survival in *Pseudomonas aeruginosa* Ventilator-Associated Pneumonia Patients

**DOI:** 10.3389/fcimb.2021.669409

**Published:** 2021-04-29

**Authors:** Xiaoling Qi, Li Zhang, Jing Xu, Zheying Tao, Xiaoli Wang, Yuzhen Qiu, Tingting Pan, Zhaojun Liu, Hongping Qu, Ruoming Tan, Jialin Liu

**Affiliations:** Department of Critical Care Medicine, Ruijin Hospital, Shanghai Jiao Tong University School of Medicine, Shanghai, China

**Keywords:** *Pseudomonas aeruginosa*, ventilator-associated pneumonia, short-chain fatty acid, lymphocyte, monocyte

## Abstract

**Background:**

We previously found that microbial disruption in *Pseudomonas aeruginosa* ventilator-associated pneumonia (PA-VAP) patients are long-lasting. Long-term microbial dysbiosis may lead to changes in metabolites. Short-chain fatty acids (SCFAs) are microbial fermentation products and show beneficial effects in patients with pneumonia. In this study, we aimed to explore the association between circulating SCFA levels and clinical outcomes in patients with PA-VAP.

**Methods:**

In this study, we analyzed SCFAs in the serum of 49 patients with PA-VAP by gas chromatography-mass spectrometry analysis. Twenty of these patients died, and 29 survived. The correlation between serum SCFAs and patient survival and immune parameters was analyzed.

**Results:**

We developed a partial least squares discriminant analysis (PLS-DA) model to examine differential SCFAs in 49 patients with PA-VAP. Among the seven SCFAs, only acetic acid was increased in non-survivors (P = 0.031, VIP > 1). Furthermore, high levels of acetic acid (>1.96ug/ml) showed increased 90-day mortality compared to low levels of acetic acid (<1.96ug/ml) in Kaplan-Meier survival analyses (P = 0.027). Increased acetic acid also correlated with reduced circulating lymphocyte and monocyte counts.

**Conclusion:**

Our study showed that increased circulating acetic acid is associated with 90-day mortality in PA-VAP patients. The decrease in lymphocytes and monocytes might be affected by acetic acid and involved in the poor prognosis.

## Introduction

*Pseudomonas aeruginosa* (PA) is a common cause of ventilator-associated pneumonia (VAP) (Sader et al., 2014; Borgatta et al., 2017). It was reported that 10%-20% of patients with PA colonization might develop VAP (Köhler et al., 2010). Only approximately 70% of PA-VAP cases can be cured despite optimizing current antimicrobial strategies (Crandon et al., 2016). In addition, patients harboring multidrug-resistant PA in the respiratory tract have a higher risk of death than those with PA colonization (Borgatta et al., 2017). These observations suggest the need for a reassessment of the treatment for PA-VAP.

In our previous study, we found that the lung microbiota of patients with PA-VAP changes significantly (Qi et al., 2018). Recent studies have also found that gut microbial dysbiosis occurs in most critically ill patients, including VAP patients (Dickson, 2016; Shimizu et al., 2018). Microbial dysbiosis in patients with VAP is characterized by decreased diversity and the disappearance of beneficial commensals (Ruppé et al., 2018), and the degree of microbial dysbiosis is related to the prognosis of the patient (Guarner and Malagelada, 2003; Shimizu et al., 2011). Multi-omic analysis has shown that microbial-derived metabolites usually drive microbiota changes in patients with a respiratory infection (Stewart et al., 2018; Bowerman et al., 2020), suggesting changes in microbial-derived metabolites might have an impact on the outcome of these patients.

Short-chain fatty acids (SCFAs) are the products of colonic microbial fermentation and can be absorbed in the gut and then drain into the circulation (Zaiss et al., 2019). They play an essential role in local microbiome balance and are likely to have a broad impact on the host immune system (Koh et al., 2016). SCFAs act on a variety of immune cells, including neutrophils, dendritic cells (Trompette et al., 2014), macrophages (Wu et al., 2020), T lymphocytes (Trompette et al., 2018), and B lymphocytes (Sanchez et al., 2020). They can reduce the recruitment and migration of dendritic cells and macrophages, and inhibit the proliferation and cytokine secretion of T cells (Gonçalves et al., 2018; Yao et al., 2020). Because SCFAs can dampen immune responses, they have a protective effect on various chronic inflammatory diseases, such as asthma and inflammatory bowel disease (Gonçalves et al., 2018).

Recent studies have shown that the anti-inflammatory properties of SCFAs could also reduce lung damage during respiratory infections (Trompette et al., 2018; Li et al., 2021). In addition, it was reported that SCFAs enhanced the effects of CD8+ T cells and macrophages to protect against respiratory infection (Trompette et al., 2018; Wu et al., 2020). These results suggested that SCFAs could also enhance the immune response. However, the role of SCFAs in the immune response was regulated by their concentration. Different from the enhancing effect of low dose-SCFAs, high-dose SCFAs can dampen innate and adaptive immune response during infections (Ciarlo et al., 2016; Sanchez et al., 2020). Besides, a recent study observed that SCFAs increase in chronic rhinosinusitis patients infected with PA (Cho et al., 2020). It was proved that SCFAs could promote pathogenically (PA and Escherichia coli) growth (Zumbrun et al., 2013; Ciarlo et al., 2016; Cho et al., 2020). Given the multiple effects of SCFAs, it is difficult to define whether they are beneficial or detrimental during bacterial infection. Clinical research is needed to clarify the role of SCFAs in those patients. Therefore, we conducted a prospective study to explore the association between serum SCFA levels and clinical outcomes in patients infected with PA.

## Methods

### Subjects

This prospective study was conducted in intensive care units (ICUs) between March 2016 and March 2020. Patients diagnosed with PA-VAP met the following criteria: (1) mechanical ventilation for more than 48 hours; (2) met at least two of the following: body temperature > 38°C or < 36°C; peripheral white blood cell count > 10 × 10^9/L or < 4 × 10^9/L; or purulent secretions; (3) new or progressive chest infiltrates, and a second evaluation was conducted for patients with underlying cardiopulmonary disease; (4) secretions of lower respiratory tract cultured PA at least +2 growth using semi-quantitative measurements. Exclusion criteria included: (1) age below 18 years; (2) pregnant women; (3) secretions of lower respiratory tract positively cultured PA before or within 48 hours of mechanical ventilation; (4) patients with structural lung disease. The clinical data collection started at hospital admission and ended at study withdrawal, discharge, or death. Healthy people were also included as a control group. Written informed consent was obtained from enrolled patients or their guardians. This study was approved by the Ruijin Hospital Ethics Committee of Shanghai Jiao Tong University School of Medicine (approval number 2012-82).

### Sample Collection

Peripheral venous blood was collected in vacutainer tubes on the first day of PA-VAP diagnosis. After blood collection, the serum of samples was collected by centrifugation (2,000 rpm, 10 min, 4°C) and stored at -80°C for SCFA and cytokine analysis.

### SCFA Analysis

For SCFA analysis, 50% H_2_SO_4_ (50 μL) was added to serum (50 μL) for acidification, and an internal standard (2-Methylvaleric acid) was used for gas chromatography-mass spectrometry (GC-MS) analysis. An Agilent 7890B gas chromatograph system coupled with an Agilent 5977B mass spectrometer fitted with a capillary column (HP-FFAP 30 m × 250 μm × 0.25 μm) was used for GC-MS analysis. An Agilent chemstation was used for chromatographic peak extraction and quantitative analysis. The standard curve of SCFA concentration was calculated according to the ratio of SCFAs and the internal standard chromatographic peak area. Seven SCFAs, acetic acid, propionic acid, isobutyric acid, butyric acid, isovaleric acid, valeric acid, and hexanoic acid were quantified.

### Immune Cell Quantification and Cytokine Detection

Immune cell quantification was conducted by Beckman Coulter LH750. Cytometric bead array (CBA) was used to detect cytokines in the serum of patients with PA-VAP. CBA kits of cytokines (IL-2, IL-6, IL-7, IL-8, IL-10, MCP-1, RANTES, VEGF) were provided by BD Biosciences (BD Biosciences, Franklin Lakes, NJ, USA). The standard was diluted to eight different concentrations, and one blank was prepared. The standard and the serum sample (50 μL) were mixed with the microspheres and incubated for 1 h at room temperature. PE-labelled cytokine antibody was added to all samples and incubated for 1 h at room temperature in the dark. All samples were washed and suspended for detection using an Accuri C6 system (BD Biosciences, Franklin Lakes, NJ, USA).

### Statistical Analysis

The difference of SCFAs was calculated using unsupervised Principal Component Analysis (PCA) and supervised Partial Least Square-Discriminant Analysis (PLS-DA). The variable projection importance (VIP) is calculated by the PLS-DA model to measure each metabolite’s impact on the classification and discrimination of each group of samples, thereby assisting screening metabolic markers. VIP values >1.0 suggest that the metabolite has significant differences between groups. VIP values and the Mann-Whitney U test were both used to assist in the screening of differential SCFAs. Clinical data were processed using SPSS version 23 (IBM Corp., Armonk, NY, USA). Differences between the two groups were tested using a two-tailed t-test, Mann-Whitney U test, or Chi-squared test as appropriate, and the significance level was set at 0.05 (2-tailed). The correlation between SCFA and clinical data and immune parameters was calculated using Spearman correlation analysis. Figures were created using GraphPad Prism version 6.0.

## Results

### Patient Characteristics

**Figure 1:**
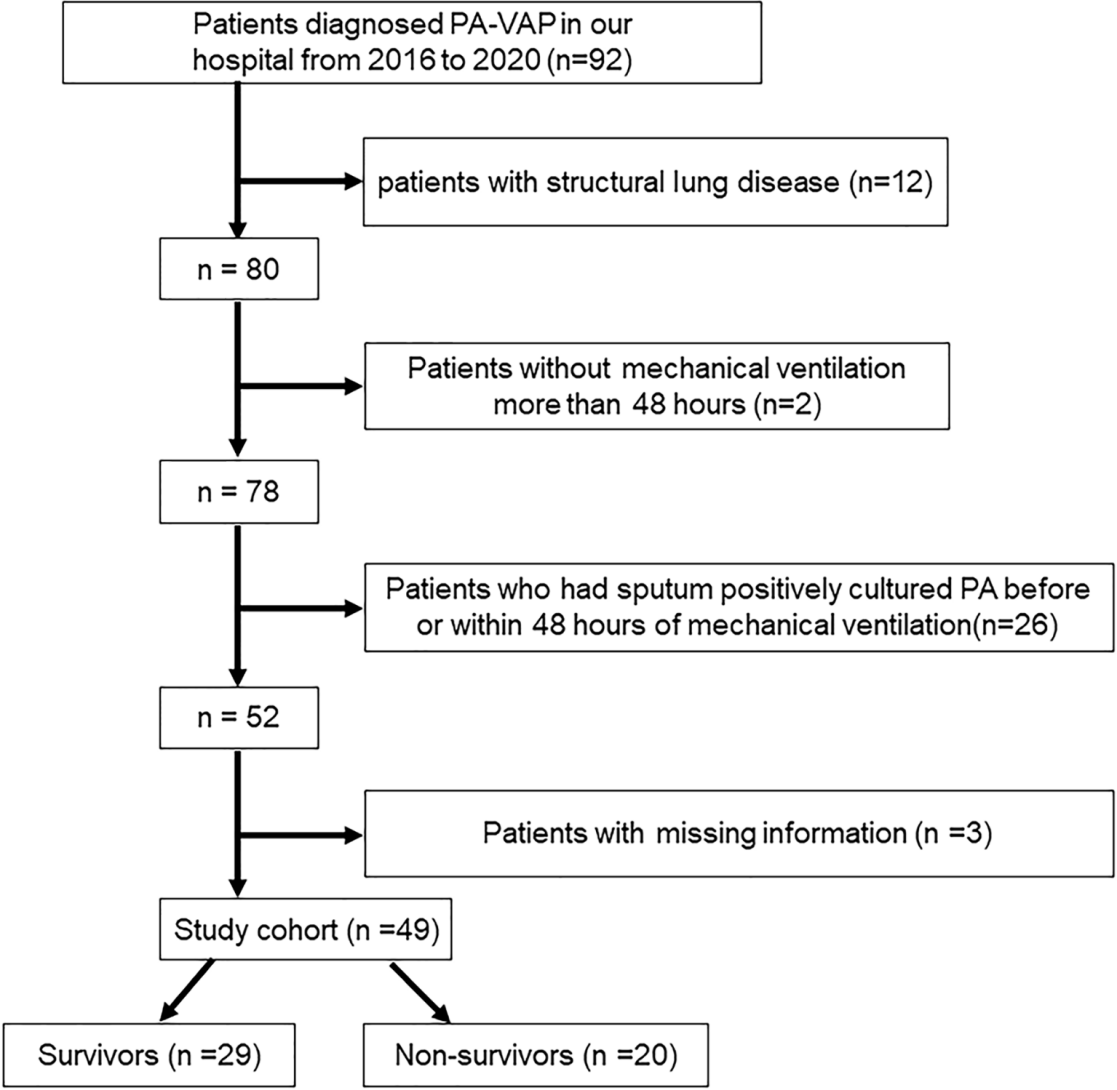
Study flow chart.

A total of 49 PA-VAP patients were included in this study, of which 20 patients died in the ICU and 29 patients survived (**Figure 1**). Baseline characteristics of all patients were collected during the first 24 hours of PA-VAP diagnosis (**Table 1**). Within the PA-VAP patients, 21 patients were admitted to the ICU due to medical diseases, 26 patients following surgery, and two patients following trauma. The type of disease at admission showed a significant difference between survivors and non-survivors (P = 0.031). The APACHE II score of non-survivors was slightly higher than that of survivors at the time of PA-VAP diagnosis (survivors vs. non-survivors, 11.48 ± 3.78 vs. 13.55 ± 4.06, P = 0.074). We also found that the number of lymphocytes and monocytes were both sharply reduced in the non-survivors (lymphocytes: survivors vs. non-survivors [1.46 ± 0.69] × 10^9/L vs. [0.92 ± 0.50] × 10^9/L, P = 0.005; monocytes: survivors vs. non-survivors 0.52 [0.39, 0.70] × 10^9/L vs. 0.32 [0.23, 0.44] × 10^9/L, P = 0.005). Among the non-survivors, 17 died of sepsis, two died of respiratory failure, and one died of circulatory failure.

**Table 1:** Baseline characteristics of PA-VAP patients.

**Characteristics**	Survivors (n=29)	Non-survivors (n=20)	P value
Demographic data			
Male, n (%)	18(62%)	13(65%)	0.834
Age, years	62.83±18.88	70.40±9.84	0.074
BMI, kg/m^2^	23.95±4.73	21.70±4.39	0.099
Current smoker, n (%)	5(17%)	5(25%)	0.508
Admission type			
Medical, n (%)	16(55%)	5(25%)	0.031
Surgical, n (%)	11(38%)	15(75%)	
Trauma, n (%)	2(7%)	0(0%)	
Charlson Comorbidity Index	2.86±1.51	3.30±1.30	0.296
Disease severity			
CPIS score	7[5. 9]	7[6, 8]	0.322
APACHE II score	11.48±3.78	13.55±4.06	0.074
SOFA score	4[2. 6.5]	5.5[3, 7]	0.154
Laboratory indicators			
White blood cell, 10^9/L	9.52[6.99, 15.11]	8.37[6.69, 12.26]	0.314
Neutrophils, 10^9/L	6.68[4.84, 11.62)	6.48[5.00, 10.40]	0.919
Lymphocytes, 10^9/L	1.46±0.69	0.92±0.50	0.005****
Monocytes, 10^9/L	0.52[0.39, 0.70]	0.32[0.23, 0.44]	0.005****
PaO2/FiO2	262.38±79.21	249.91±90.06	0.611
Cause of death			
Sepsis	/	17	
Respiratory failure	/	2	
Circulatory failure	/	1	

BMI, body mass index; SOFA, Sequential Organ Failure Assessment; APACHE, Acute Physiology and Chronic Health Evaluation; CPIS, clinical pulmonary infection score.

### Circulating Acetic Acid Change in Relation to Survival

**Figure 2:**
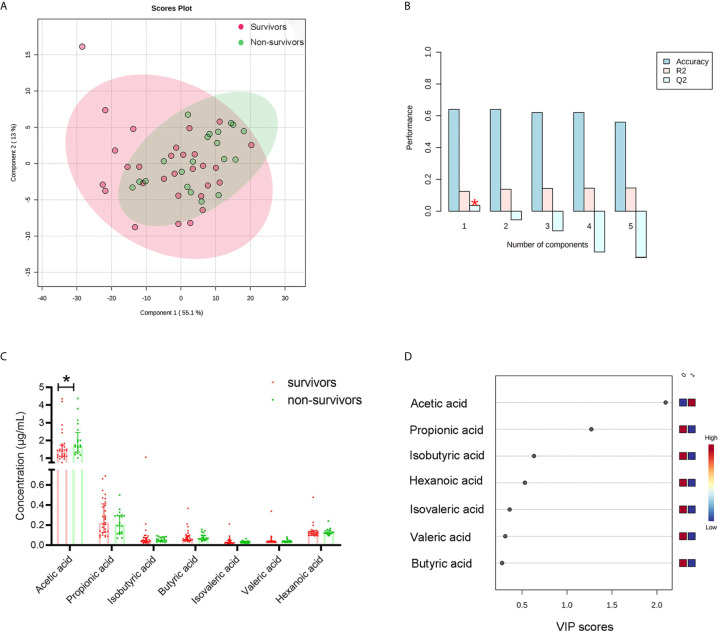
Differential SCFA analysis between survivors and non-survivors with PA-VAP. **(A)** Supervised clustering conducted by partial least squares discriminant analysis (PLS-DA) revealed that the SCFAs were different between the two groups. **(B)** Cross-validation test for PLS-DA model. **(C)** Mann Whitney analysis demonstrated that only acetic acid was significantly different between the two groups. **(D)** The Variable Importance in Projection score of acetic acid and propionic acid were both > 1. PA-VAP, *Pseudomonas aeruginosa* ventilator-associated pneumonia; SCFAs, short chain fatty acids; *P < 0.05.

Ten healthy people were recruited as control group. All of SCFAs significantly decreased in PA-VAP patients compared to healthy people (**Supplementary Table S1** and **Supplementary Figure S1**). To explore the impact of circulating SCFAs on survival, all SCFAs were subjected to supervised PLS-DA and unsupervised PCA analysis. Samples from the survivors and non-survivors showed significant differences in the PLS-DA model but not in the PCA analysis (**Figures 2A, B** and **Supplementary Figure S2**). VIP scores were also calculated to identify differential SCFAs. The VIP scores of acetic acid and propionic acid were both > 1, while only the P value of acetic acid was < 0.05 (survivors vs. non-survivors 1.69[1.51,2.88] vs. 1.73[1.46,2.60], P = 0.031) (**Figures 2C, D**). This suggests that decreased circulating acetic acid is associated with survival.

### 90-Day Mortality Was Associated With Circulating Acetic Acid

**Figure 3:**
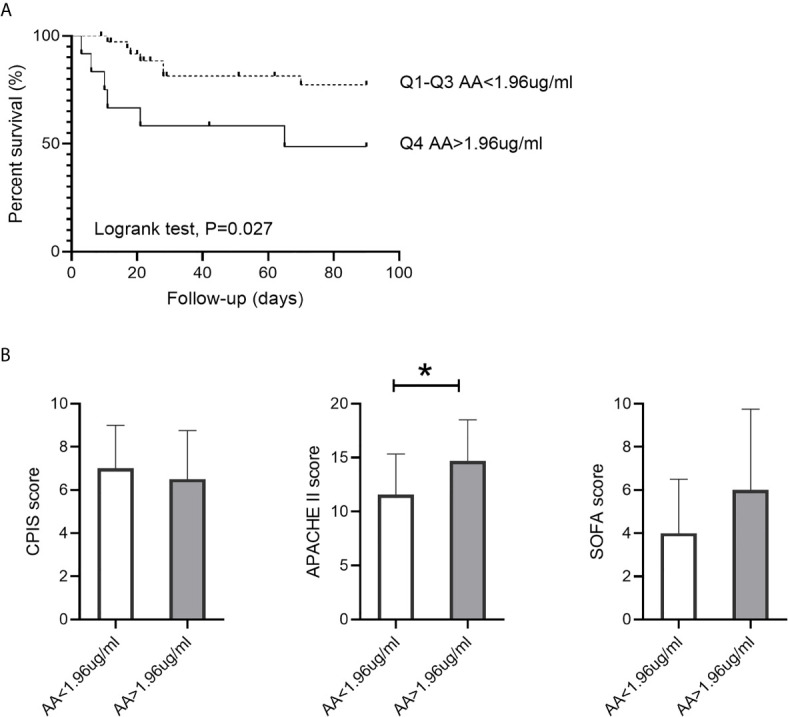
Association between circulating acetic acid concentration and 90-day mortality. **(A)** Kaplan-Meier survival analysis grouped by acetic acid quartile in PA-VAP patients. **(B)** Comparison of the CPIS score, APACHE II score, and SOFA score between high acetic acid concentration group (Q4, >1,96ug/ml) and low acetic acid concentration group (Q1-Q3, <1.96ug/ml) in PA-VAP patients. PA-VAP, *Pseudomonas aeruginosa* ventilator-associated pneumonia; AA, acetic acid; SOFA, Sequential Organ Failure Assessment; APACHE, Acute Physiology and Chronic Health Evaluation; CPIS, clinical pulmonary infection score; *P < 0.05.

As shown in **Figure 2**, only acetic acid was increased in non-survivors among the seven circulating SCFAs. We, therefore, performed Kaplan-Meier survival analyses according to the level of acetic acid. Patients with acetic acid in the fourth quartile(Q4, >1.96 ug/ml) versus those in the first to third quartiles(Q1-Q3, <1.96ug/ml) showed significantly higher 90-day mortality(P = 0.027) (**Figure 3A**). The degree of disease severity of all patients was evaluated on the day of diagnosis of PA-VAP. Among the three evaluation systems, we observed higher APACHE II scores in patients with acetic acid in the fourth quartile compared to the first to third quartiles (AA<1.96ug/ml vs. AA>1.96ug/ml 11.57 ± 3.78 vs. 14.67 ± 3.85, P = 0.018). CPIS score (P = 0.869) and SOFA score (P = 0.053) were not significantly different between the two groups (**Figure 3B**). We also conducted Kaplan-Meier survival analyses according to the level of other six SCFAs. Conversely, compared with the first to third quartiles (Q1-Q3, <0.333ug/ml), propionic acid in the fourth quartile (Q4,> 0.333ug/ml) showed a trend of lower 90-day mortality) (P = 0.079) (**Supplementary Figure S3**). There is no significant correlation between the levels of the other five SCFAs and the 90-day mortality rate of PA-VAP patients) (**Supplementary Figure S3**).

### Correlation of Circulating Acetic Acid and Host Immune Response

**Figure 4:**
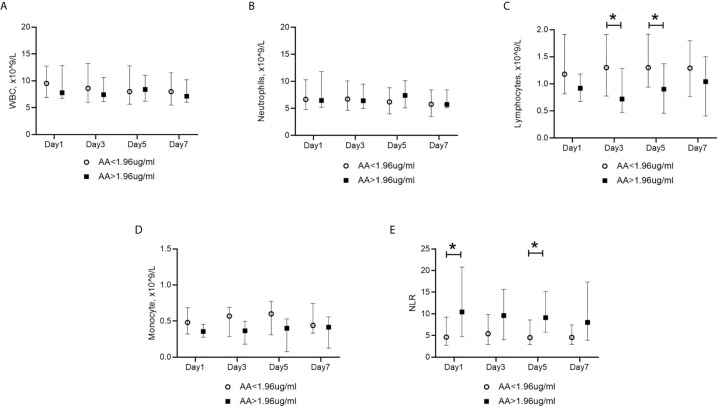
Dynamic changes in blood immune cells in PA-VAP patients. Grouping depended on the acetic acid quartile. **(A, B)** Similar levels of WBCs and neutrophils in the high acetic acid concentration group (Q4, >1,96ug/ml) and low acetic acid concentration group (Q1-Q3, <1.96ug/ml) within the 7-day observation period. **(C, D)** Higher levels of lymphocytes and monocytes were found in the high acetic acid concentration group (Q4, >1,96ug/ml). **(E)** lower level of NLR was found in the high acetic acid concentration group (Q4, >1,96ug/ml). PA-VAP, *Pseudomonas aeruginosa* ventilator-associated pneumonia; AA, acetic acid; WBC, white blood cell; NLR, neutrophil-lymphocyte ratio; *P < 0.05.

As shown in **Table 1**, non-survivors had lower blood lymphocyte and monocyte counts. We, therefore, performed a Spearman correlation analysis between acetic acid and circulating immune parameters. Lymphocyte and monocyte counts were both negatively correlated with acetic acid (**Table 2** and **Supplementary Figures S4A, B**). However, no significant correlation was observed between white blood cell count, neutrophil count, neutrophil-lymphocyte ratio, and acetic acid (**Table 2**). We also explored the dynamic changes in immune cells within seven days after the diagnosis of PA-VAP, grouped by acetic acid quartile. White blood cell and neutrophil counts showed no significant difference between the two groups (**Figures 4A, B**), while patients with acetic acid in the fourth quartile (Q4, >1.96 ug/ml) showed continuous lower lymphocyte and monocyte counts compared to those in the first to third quartiles (Q1-3, <1.96 ug/ml) (**Figures 4C, D**). We also found that patients with acetic acid in the fourth quartile (Q4, >1.96 ug/ml) showed a continuously higher trend in the neutrophil-lymphocyte ratio (**Figure 4E**). We then asked whether circulating acetic acid was associated with systemic inflammation, as measured on the diagnosis day. Only IL-2 was significantly negatively correlated with circulating acetic acid (P = 0.022, R = -0.455) (**Supplementary Table S2** and **Supplementary Figure S4C**). On the other hand, we conducted a Spearman correlation analysis to explore the correlation between acetic acid and clinical parameters. We found that platelet counts were negatively correlated with circulating acetic acid (P = 0.005, R = -0.397) (**Supplementary Table S3**).

**Table 2:** Correlation between acetic acid and circulating immune cells in PA-VAP patients.

Immune parameters	R	P
White blood cell, 10^9/L	-0.107	0.46
Neutrophils, 10^9/L	0.025	0.86
Lymphocytes, 10^9/L	-0.288****	****0.045
Neutrophils/Lymphocytes	0.255	0.077
Monocyte, 10^9/L	-0.367****	0.009****

### Effects of ICU Related Factors on Circulating Acetic Acid Concentration

**Figure 5:**
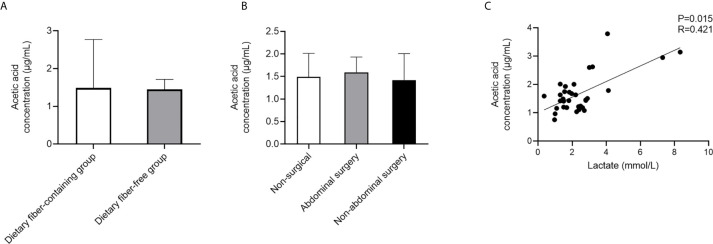
Analysis of factors affecting acetic acid level in PA-VAP patients. **(A)** The concentration of acetic acid was not significantly different between the dietary fiber-containing and dietary fiber-free groups. **(B)** Level of acetic acid was similar among the no surgery group, abdominal surgery group, and non-abdominal surgery group. **(C)** Serum acetic acid showed a significant positive correlation with serum lactate in PA-VAP patients without dietary fiber intake. PA-VAP, *Pseudomonas aeruginosa* ventilator-associated pneumonia.

Acetic acid is produced by intestinal microbiota digesting specific dietary components, mainly dietary fiber. Within the whole cohort, 36 (73.5%) patients received enteral nutrition, two(4.1%) patients received parenteral nutrition, six (12.2%) patients fasted, and five (10.2%) patients received a normal diet. According to the composition of enteral nutrition, we divided the patients who received enteral nutrition into a dietary fiber-containing group and a dietary fiber-free group. Acetic acid showed similar concentrations in the dietary fiber-containing and dietary fiber-free groups (P=0.89) (**Figure 5A**). Considering the impact of abdominal surgery on intestinal microbiota, we divided the whole cohort into no surgery, abdominal surgery, and non-abdominal surgery groups to explore the effect of surgery on circulating acetic acid. Acetic acid showed no significant difference among the three groups(P = 0.94) (**Figure 5B**). It is reported that lactate is also a precursor of SCFAs, so we conducted Spearman correlation analysis to observe the association between lactate and SCFAs. In PA-VAP patients, there was a significant positive correlation between isobutyric acid and serum lactate (P=0.035, R=0.334), while the other six SCFAs have no significant correlation with lactate levels (**Supplementary Table S4**). Considering that dietary fiber can affect the level of SCFAs, we re-analyzed the correlation between lactate levels and SCFAs in PA-VAP patients without dietary fiber intake. We found that acetic acid, butyric acid, and isobutyric acid showed a significant positive correlation with lactate levels (acetic acid: P=0.015, R=0.421) (**Supplementary Table S4** and **Figure 5C**).

## Discussion

SCFAs are the main products of the microbial fermentation of dietary fiber and can have various effects on host physiology (Koh et al., 2016). PA-VAP is a common complication in critically ill patients with obvious “imbalanced microbiota” and “imbalanced immunity”. However, little is known about the relationship between SCFAs and clinical outcomes in PA-VAP patients. In this study, we found that high serum acetic acid levels were associated with disease severity and poor prognosis in PA-VAP patients. We also observed that high acetic acid levels might be related to immunosuppression, which mainly showed a negative correlation between acetic acid concentrations and lymphocyte counts and monocyte counts. Previous studies have also shown that persistent low circulating lymphocytes and monocytes are associated with a poor prognosis of lung infection (Ceccato et al., 2019; Feng et al., 2019). These findings indicate that high levels of serum acetic acid may have detrimental effects on PA-VAP patients.

Firstly, we found that serum acetic acid increased in non-surviving PA-VAP patients, and the increased serum acetic acid was associated with disease severity and mortality. Although it is generally believed that SCFAs have various benefits rather than detriments, SCFAs have not shown obvious beneficial effects on bacterial infections. SCFAs could inhibit cytokines and nitric oxide production by monocytes/macrophages and not improve the survival of pulmonary-sepsis (Ciarlo et al., 2016). Furthermore, recent studies found that excessive SCFAs have detrimental effects on the host (Zumbrun et al., 2013; Cho et al., 2020). It has been reported that a high-fiber diet (producing more SCFAs) promotes pathogenic Escherichia coli O157:H7 strain colonization and increases mortality (Zumbrun et al., 2013). In addition, SCFAs, especially acetic acid, could also promote the proliferation of PA. The high concentration of acetic acid in sinusitis mucus was a risk factor for chronic rhinosinusitis with PA infection (Cho et al., 2020). On the other hand, some clinical studies also found that SCFAs may be detrimental (Kurilshikov et al., 2019; Behary et al., 2021). It was reported that SCFAs were associated with poorer clinical outcomes in hepatocellular carcinoma patients (Behary et al., 2021). Since most of the protective effects of SCFAs have been found in mice, more clinical studies are needed to clarify the role of SCFAs in bacterial pneumonia.

Then we found that PA-VAP patients with high acetic acid levels had lower numbers of monocytes and lymphocytes in the peripheral blood compared to those with low levels of acetic acid. Acetic acid is an inhibitor of histone deacetylases (HDACs) and plays an essential role in immunosuppression (Koh et al., 2016). It was reported that acetic acid damages the T-dependent immune responses and prevents the activation of effector B cells (Azizov et al., 2020). Acetic acid can also directly inhibit the production of cytokines by human monocytes under inflammatory stimuli (Cox et al., 2009; Ang et al., 2016). The immunosuppressive effect of acetic acid on human monocytes was mediated by P38 phosphorylation mediated by G protein-coupled receptors 41/G protein-coupled receptors 43 (Ang et al., 2016). However, the immunosuppressive effect of acetic acid on human monocytes did not appear in mice (Ang et al., 2016). These findings suggest that additional clinical studies are needed to investigate the role of acetic acid. On the other hand, it is worth noting that different concentrations of SCFAs can induce different effects. High-dose SCFAs reduced the expression of AID and Blimp1 in B cells, inhibit class-switch DNA recombination, somatic hypermutation, and plasma cell differentiation, thereby inhibiting local and systemic antibody responses in mice. At the same time, low-dose SCFAs could promote local and systemic antibody responses (Sanchez et al., 2020). As is known, butyric acid can provide energy for colon cells and prevent infection. However, butyric acid at physiological concentration can inhibit intestinal stem cells’ proliferation (Kaiko et al., 2016). In this study, we found that high concentration of acetic acid may have immunosuppressive effects in PA-VAP patients. It suggested that high levels of acetic acid may be detrimental to patients who have already developed PA-VAP.

We also found that patients with high levels of serum acetic acid had higher mortality. Those patients showed a continuously lower level of monocytes and lymphocytes and a higher neutrophil-lymphocyte ratio in the peripheral blood. As reported, continuous lymphocytopenia and a high neutrophil-lymphocyte ratio can predict mortality in patients with ICU-acquired pneumonia (Akilli et al., 2014; Ceccato et al., 2019; Feng et al., 2019). On the other hand, even with appropriate antibiotic therapy, lymphocytopenia occurring during the day of diagnosis may increase the risk of treatment failure in VAP patients (Gursel et al., 2008). IL-2 plays an important role in promoting lymphocyte proliferation. It was reported that the severity of pneumonia in critically ill patients was associated with IL-2-induced lymphocytopenia (Shi et al., 2020). Consistent with the effects of IL-2, we found that IL-2 concentration and the number of lymphocytes were both negatively related to serum acetic acid, which suggests that serum acetic acid may decrease the number of lymphocytes via the IL-2 signaling pathway.

SCFAs are the products of colonic microbial fermentation. Acetic acid, propionate, and butyrate are the major SCFAs. Butyrate is an important energy source for colonocytes and is locally consumed in the colon, while acetic acid and propionate are absorbed into the peripheral circulation. SCFAs are regulated mainly by diet and gut microbiota (Koh et al., 2016). The microbiota of critically ill patients changes dramatically, including decreasing microbial diversity, loss of commensal, and potentially pathogenic species (McDonald et al., 2016). Interventions in the ICU, such as antibiotics, invasive devices, and enteral nutrition, could profoundly affect the microbiota. In this study, we found that the intake of fiber and gastrointestinal surgery had no significant effects on serum acetic acid level. This may be relevant to the different dietary fiber intakes. As is known, lactate can also be converted into SCFAs (Koh et al., 2016). In this study, we observed that levels of acetic acid and butyric acid showed a significant positive correlation with lactate levels in PA-VAP patients without dietary fiber intake. It suggested that the elevated acetic acid in the non-survivors with PA-VAP may be related to excessive lactate.

This study had some limitations. Firstly, the sample size was limited. Secondly, the gut microbiota, fecal SCFAs, and intestinal permeability markers were not measured simultaneously in this study. These parameters may provide a better understanding of why circulating acetic acid levels increased in non-survivors. Thirdly, as there was no record of daily dietary fiber intake in PA-VAP patients before sampling, a prospective cohort should be conducted to identify the causal relationship between dietary fiber and circulating acetic acid levels in PA-VAP patients. Fourthly, since the antibiotic strategies of PA-VAP patients showed significant interpersonal differences, we cannot perform statistical analysis to obtain the impact of antibiotics on the level of SFCAs. Although we have found that acetic acid may be related to immunosuppression in PA-VAP patients, further mechanical experiments are needed to verify this association.

In summary, the level of acetic acid in serum was negatively correlated with lymphocytes and monocytes in PA-VAP patients. These results suggest that higher serum acetic acid levels in patients with PA-VAP may lead to immunosuppressive and dampen anti-infection immunity responses, which may increase mortality.

## Data Availability Statement

The raw data supporting the conclusions of this article will be made available by the authors, without undue reservation.

## Ethics Statement

The studies involving human participants were reviewed and approved by Ruijin Hospital Ethics Committee Shanghai Jiao Tong University School of Medicine. The patients/participants provided their written informed consent to participate in this study.

## Author Contributions

JL, XQ, and RT contributed to the study design. ZL, XW, TP, and HQ contributed to the subject recruitment and the sample collection. XQ, LZ, JX, and ZT, performed the experiments. JL, XQ, and YQ contributed to data analysis. JL, XQ, RT, and LZ contributed to data interpretation. All authors contributed to the article and approved the submitted version.

## Funding

This study was supported by the National Natural Science Foundation of China (81770005) and (81970005) awarded to JL, Medical-engineering Cross Foundation of Shanghai Jiao Tong University grant “2019-nCoV research project” (YG2020YQ30) awarded to JL, Talent development project for Three-years action plan of Shanghai public health system construction (GWV-10.2-XD03) awarded to JL, National Key R&D Program of China (2017YFC1309700, 2017YFC1309705) awarded to HQ, National Natural Science Foundation of China(81801885) awarded to TP. Shanghai Sailing Program (18YF1413800) awarded to TP.

## Conflict of Interest

The authors declare that the research was conducted in the absence of any commercial or financial relationships that could be construed as a potential conflict of interest.

## Supplementary Material

Supplementary Figure 1 Unsupervised clustering was conducted by Principal Component Analysis (PCA) in the whole cohort. The composition of serum SCFAs in PA-VAP patients was significantly different from that of healthy people. PA-VAP, *Pseudomonas aeruginosa* ventilator-associated pneumonia; SCFAs, short chain fatty acids.

Supplementary Figure 2 Unsupervised clustering conducted by Principal Component Analysis (PCA) revealed that the level of SCFAs were similar between surviving PA-VAP patients and non-surviving PA-VAP patients. PA-VAP, *Pseudomonas aeruginosa* ventilator-associated pneumonia; SCFAs, short chain fatty acids.

Supplementary Figure 3 Association between circulating SCFAs concentration and 90-day mortality. A Kaplan-Meier survival analysis grouped by propionic acid **(A)**, isobutyric acid **(B)**, butyric acid **(C)**, isovaleric acid **(D)**, valeric acid **(E)**, and hexanoic acid **(F)** quartile in PA-VAP patients. PA-VAP, *Pseudomonas aeruginosa* ventilator-associated pneumonia; SCFAs, short chain fatty acids.

Supplementary Figure 4 Correlation between acetic acid and circulating immune cells **(A, B)** and IL-2 **(C)** in PA-VAP patients. PA-VAP, *Pseudomonas aeruginosa* ventilator-associated pneumonia.

## Abbreviations

PA-VAP, *Pseudomonas aeruginosa* ventilator-associated pneumonia; SCFAs, short-chain fatty acids; PCA, Principal Component Analysis; PLS-DA, partial least squares discriminant analysis; VAP, ventilator-associated pneumonia; ICUs, intensive care units; CBA, cytometric bead array; VIP, Variable Importance in Projection; HDACs, histone deacetylases; BMI, body mass index; SOFA, Sequential Organ Failure Assessment; APACHE, Acute Physiology and Chronic Health Evaluation; CPIS, clinical pulmonary infection score; MCP-1, Monocyte chemotactic protein-1; RANTES, regulated upon activation of normal T cells expressed and secreted; VEGF, vascular endothelial growth factor.
