# Supplementary figures and images for: Association of Increased Circulating Acetic Acid With Poor Survival in Pseudomonas aeruginosa Ventilator-Associated Pneumonia Patients
Source: Front Cell Infect Microbiol. 2021 Apr 29;11:669409. doi: 10.3389/fcimb.2021.669409 (PMC8117141; doi:10.3389/fcimb.2021.669409)

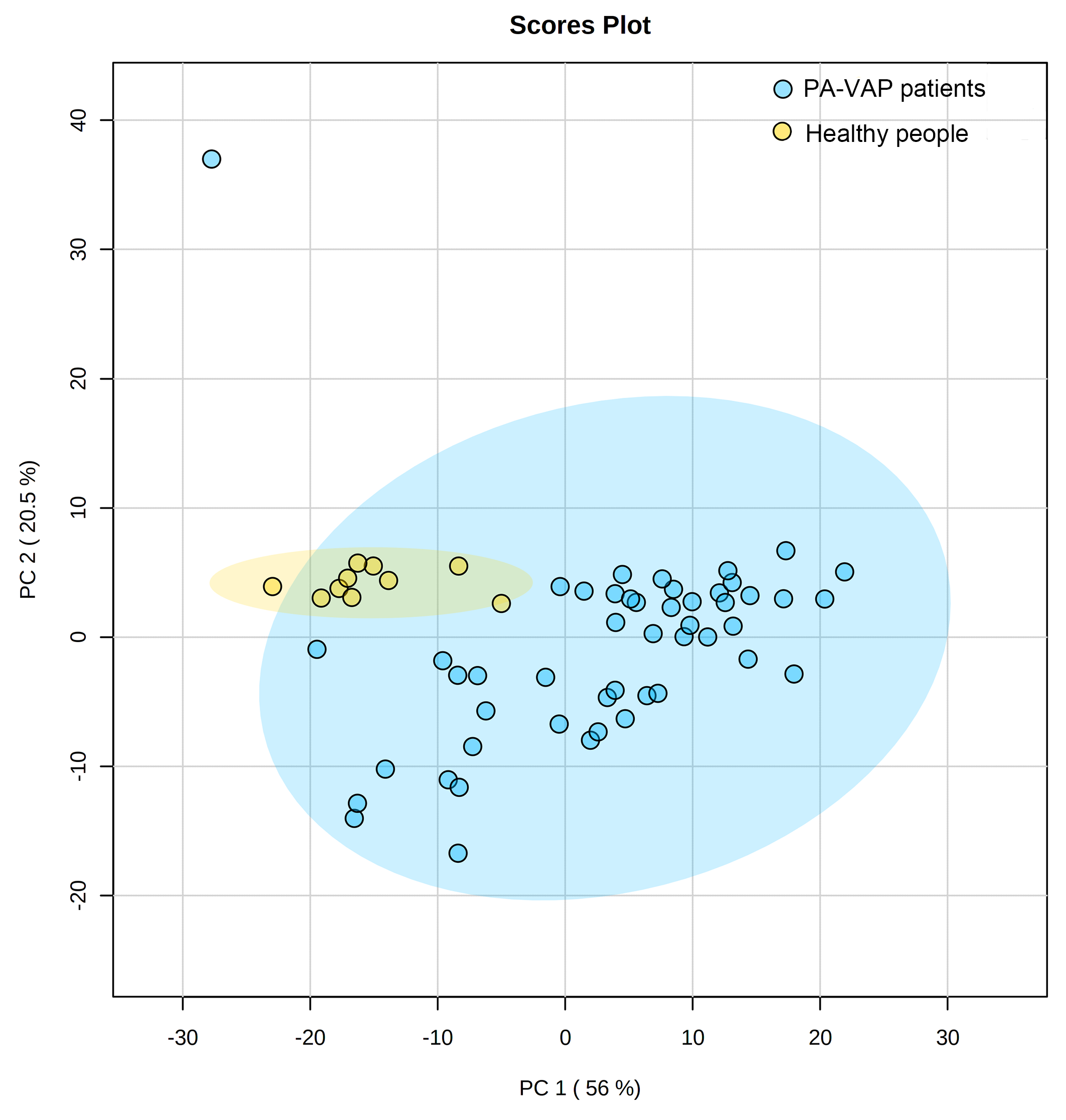

Supplement: Supplementary Figure 1 — Unsupervised clustering was conducted by Principal Component Analysis (PCA) in the whole cohort. The composition of serum SCFAs in PA-VAP patients was significantly different from that of healthy people. PA-VAP, Pseudomonas aeruginosa ventilator-associated pneumonia; SCFAs, short chain fatty acids. [file Image_1.tif]

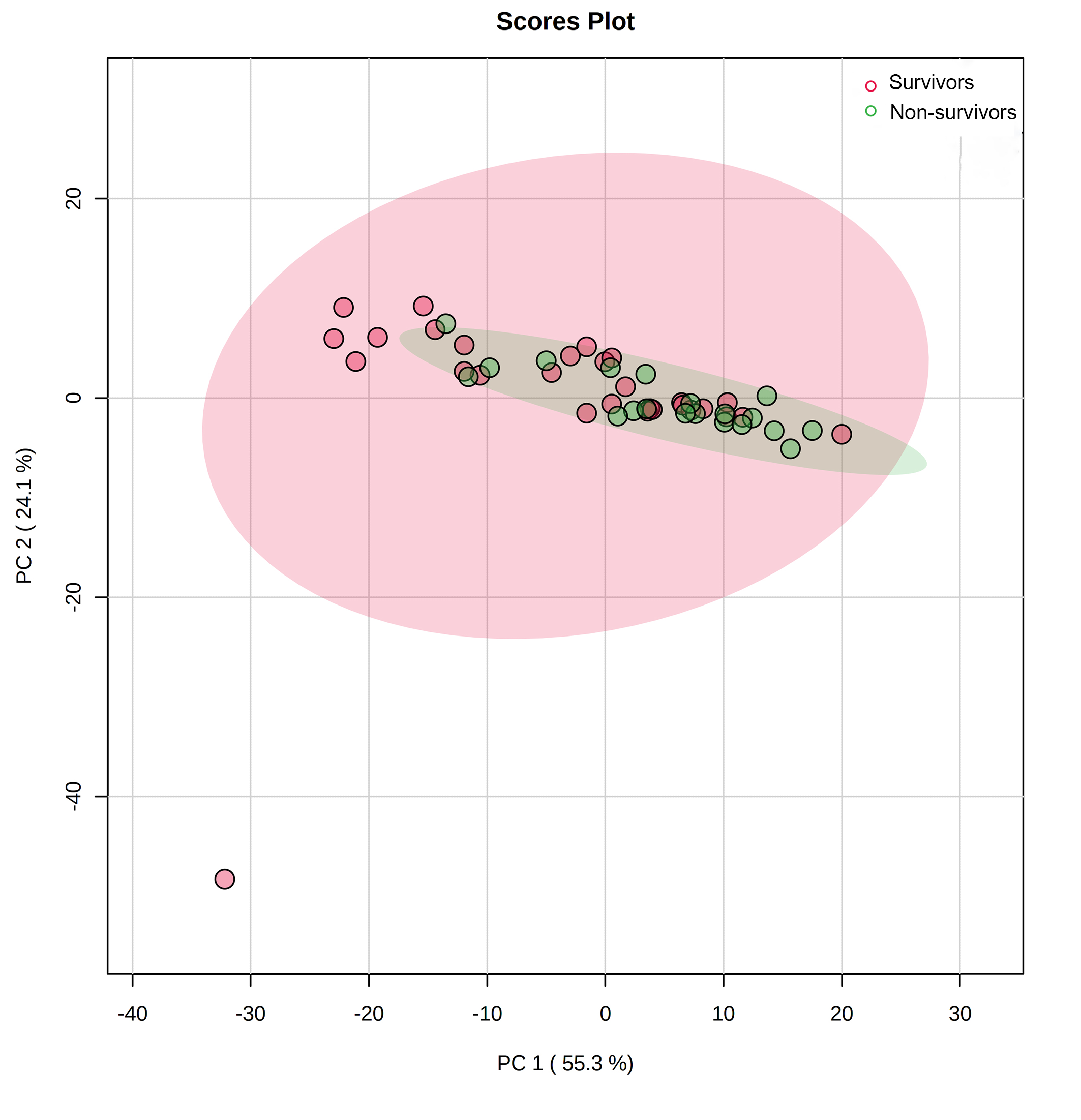

Supplement: Supplementary Figure 2 — Unsupervised clustering conducted by Principal Component Analysis (PCA) revealed that the level of SCFAs were similar between surviving PA-VAP patients and non-surviving PA-VAP patients. PA-VAP, Pseudomonas aeruginosa ventilator-associated pneumonia; SCFAs, short chain fatty acids. [file Image_2.tif]

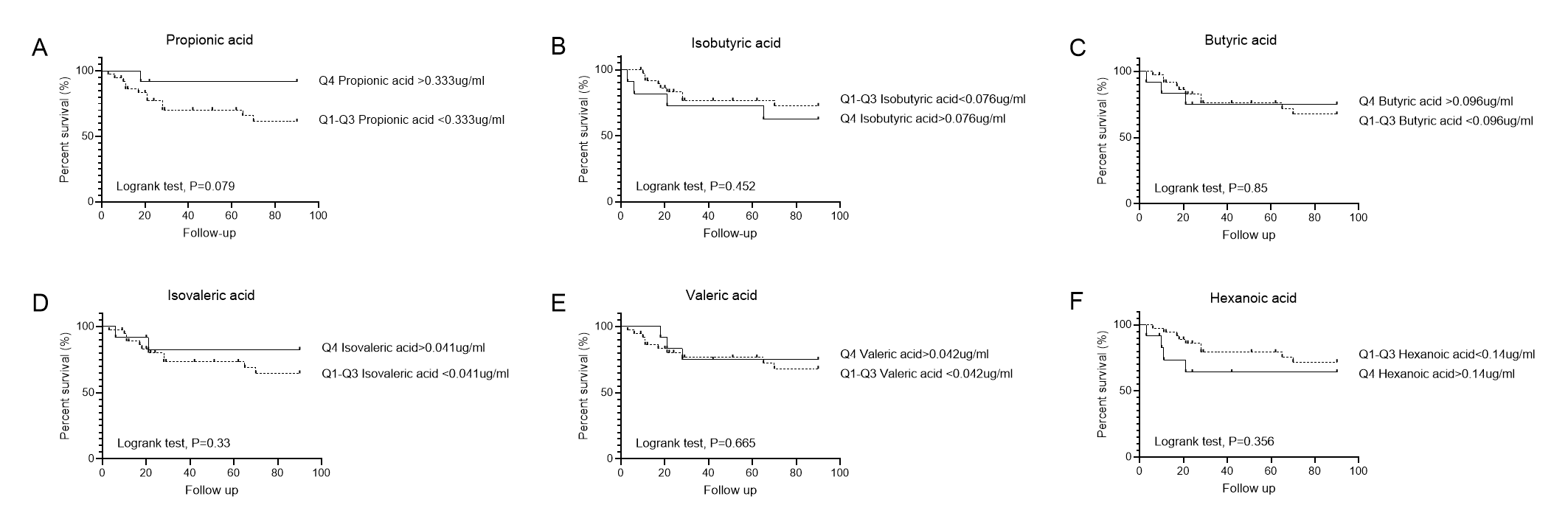

Supplement: Supplementary Figure 3 — Association between circulating SCFAs concentration and 90-day mortality. A Kaplan-Meier survival analysis grouped by propionic acid (A), isobutyric acid (B), butyric acid (C), isovaleric acid (D), valeric acid (E), and hexanoic acid (F) quartile in PA-VAP patients. PA-VAP, Pseudomonas aeruginosa ventilator-associated pneumonia; SCFAs, short chain fatty acids. [file Image_3.tif]

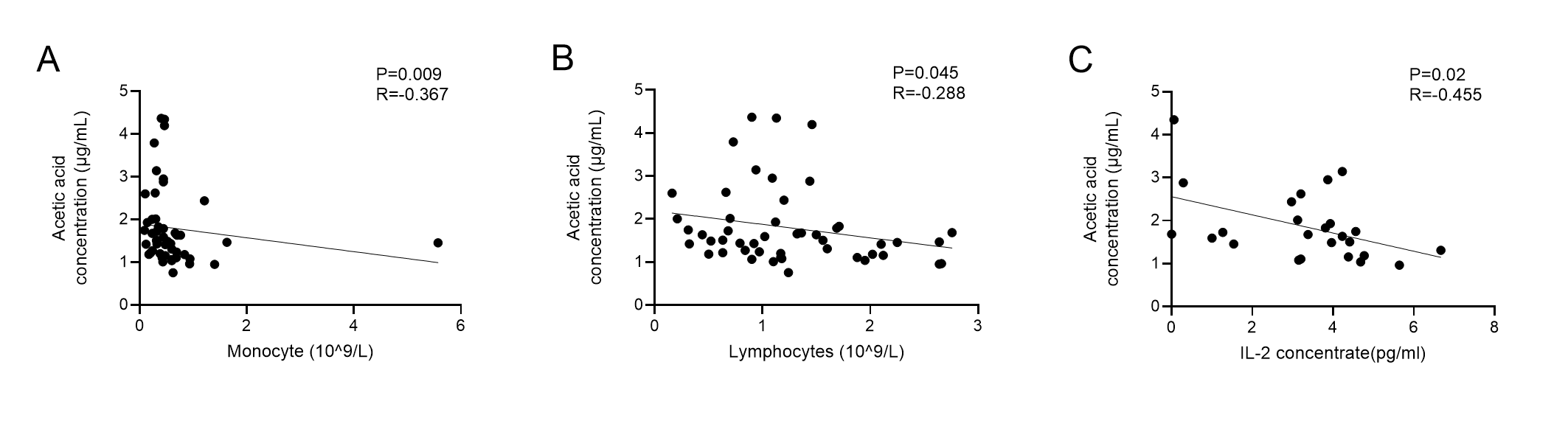

Supplement: Supplementary Figure 4 — Correlation between acetic acid and circulating immune cells (A, B) and IL-2 (C) in PA-VAP patients. PA-VAP, Pseudomonas aeruginosa ventilator-associated pneumonia. [file Image_4.tif]
