# Supplementary material for: Association of Increased Circulating Acetic Acid With Poor Survival in Pseudomonas aeruginosa Ventilator-Associated Pneumonia Patients
Source: Front Cell Infect Microbiol. 2021 Apr 29;11:669409. doi: 10.3389/fcimb.2021.669409 (PMC8117141; doi:10.3389/fcimb.2021.669409)
Supplement: Supplementary file 5 [file Table_1.docx]

Table S1 SCFAs significantly decreased in PA-VAP patients compared to healthy people.

|  | Healthy  (N=10) | PA-VAP patients  (N=49) | P value |
| --- | --- | --- | --- |
| Acetic acid | 2.69[2.35,2.94] | 1.50[1.20,1.97] | 0.001 |
| Propionic acid | 0.43[0.38,0.47] | 0.22[0.12,0.33] | 0.001 |
| Butyric acid | 0.26[0.22,0.26] | 0.065[0.051,0.096] | <0.001 |
| Isobutyric acid | 0.21[0.18,0.28] | 0.049[0.033,0.076] | <0.001 |
| Valeric acid | 0.26[0.21,0.27] | 0.034[0.030,0.042 | <0.001 |
| Isovaleric acid | 0.19[0.18,0.20] | 0.027[0.021,0.041] | <0.001 |
| Hexanoic acid | 0.48[0.42,0.57] | 0.12[0.11,0.14] | <0.001 |

SCFA short chain fatty acid; PA-VAP *Pseudomonas aeruginosa* ventilator-associated pneumonia
