# Supplementary material for: Association of Increased Circulating Acetic Acid With Poor Survival in Pseudomonas aeruginosa Ventilator-Associated Pneumonia Patients
Source: Front Cell Infect Microbiol. 2021 Apr 29;11:669409. doi: 10.3389/fcimb.2021.669409 (PMC8117141; doi:10.3389/fcimb.2021.669409)
Supplement: Supplementary file 6 [file Table_2.docx]

Table S2 Correlation between acetic acid and cytokines in PA-VAP patients.

| cytokines | R | P |
| --- | --- | --- |
| IL-2, pg/ml | **-0.455** | **0.022** |
| IL-6, pg/ml | 0.111 | 0.59 |
| IL-7, pg/ml | -0.124 | 0.55 |
| IL-8, pg/ml | 0.287 | 0.16 |
| IL-10, pg/ml | 0.248 | 0.23 |
| MCP-1, pg/ml | 0.06 | 0.76 |
| RANTES, pg/ml | -0.09 | 0.66 |
| VEGF, pg/ml | 0.09 | 0.65 |

PA-VAP *Pseudomonas aeruginosa* ventilator-associated pneumonia; MCP-1 Monocyte chemotactic protein-1, RANTES Regulated upon activiation normal T cell expressed and secreted, VEGF Vascular endothelial growth factor.
