# Supplementary material for: Association of Increased Circulating Acetic Acid With Poor Survival in Pseudomonas aeruginosa Ventilator-Associated Pneumonia Patients
Source: Front Cell Infect Microbiol. 2021 Apr 29;11:669409. doi: 10.3389/fcimb.2021.669409 (PMC8117141; doi:10.3389/fcimb.2021.669409)
Supplement: Supplementary file 7 [file Table_3.docx]

Table S3 Correlation between acetic acid and clinical parameters in PA-VAP patients.

| Clinical parameters | R | P |
| --- | --- | --- |
| PH | 0.052 | 0.724 |
| PaO2/FiO2 | 0.07 | 0.633 |
| Hematocrit, 10^9/L | -0.178 | 0.222 |
| Platelet,×10^9/L | **-0.397** | **0.005** |
| Total bilirubin | 0.108 | 0.458 |
| Bile acid | 0.207 | 0.154 |

PA-VAP *Pseudomonas aeruginosa* ventilator-associated pneumonia
