# Supplementary material for: Association of Increased Circulating Acetic Acid With Poor Survival in Pseudomonas aeruginosa Ventilator-Associated Pneumonia Patients
Source: Front Cell Infect Microbiol. 2021 Apr 29;11:669409. doi: 10.3389/fcimb.2021.669409 (PMC8117141; doi:10.3389/fcimb.2021.669409)
Supplement: Supplementary file 8 [file Table_4.docx]

Table S4. Correlation between SCFAs and lactate in PA-VAP patients.

| SCFAs | All PA-VAP patients | | PA-VAP patients without dietary fiber intake | |
| --- | --- | --- | --- | --- |
|  | R | P | R | P |
| Acetic acid | 0.188 | 0.245 | **0.421** | **0.015** |
| Propionic acid | 0.202 | 0.211 | 0.245 | 0.170 |
| Butyric acid | 0.285 | 0.074 | **0.401** | **0.018** |
| Isobutyric acid | **0.334** | **0.035** | **0.455** | **0.0079** |
| Valeric acid | 0.164 | 0.313 | 0.239 | 0.18 |
| Isovaleric acid | 0.184 | 0.255 | 0.308 | 0.081 |
| Hexanoic acid | 0.148 | 0.363 | 0.242 | 0.17 |

SCFA short chain fatty acid; PA-VAP *Pseudomonas aeruginosa* ventilator-associated pneumonia
